# Current News

**Published:** 1893-01

**Authors:** 


### Current News.

### RECENT PATENTS.

A LIST of recent patents, reported specially for the INTERNATIONAL DENTAL JOURNAL:

485,280. Amalgam for Dentists' Use. Gustav Juterbock, Berlin, Germany. Filed January 4, 1892.

485,383. Dental Chair. Samuel T. Henkle, Baltimore, Md., assignor to the Henkle Dental Chair Company, Chicago, Ill. Filed January 22, 1891.

485,609. Mouth-holding Apparatus for Dentists and Surgeons. Howard M. Casebeer, Lincoln, Neb. Filed March 18, 1892.

486,112. Dental Spacer. Nathaniel Kuns, Fairbury, Neb. Filed April 11, 1892.

